# A handy and accessible tool for identification of Sn(II) in toothpaste

**DOI:** 10.1038/s41598-022-06299-0

**Published:** 2022-02-10

**Authors:** Shampa Kundu, Khai-Nghi Truong, Shrabani Saha, Kari Rissanen, Prithidipa Sahoo

**Affiliations:** 1 Department of Chemistry, Visva-Bharati University, Santiniketan, W.B. 731235 India; 2 Department of Chemistry, University of Jyvaskyla, Survontie 9 B, P.O. Box 35, 40014 Jyväskylä, Finland

**Keywords:** Environmental sciences, Chemistry

## Abstract

An easily accessible colorimetric probe, a carbazole–naphthaldehyde conjugate (**CNP**), was successfully prepared for the selective and sensitive recognition of Sn(II) in different commercially-available toothpaste and mouth wash samples. The binding mechanism of **CNP** for Sn^2+^ was confirmed by UV–Vis, ^1^H, and ^13^C NMR titrations. The proposed sensing mechanism was supported by quantum chemical calculations. Selective detection of Sn(II) in the nanomolar range (85 nM), among other interfering metal ions, makes it exclusive. Moreover, Sn^2+^ can be detected with a simple paper strip from toothpaste, which makes this method handy and easily accessible. The potential application of this system for monitoring Sn^2+^ can be used as an expedient tool for environmental and industrial purposes.

## Introduction

The utilization of tin has been established for thousands of years and the most commonly used for the manufacturing of bronze, which is a tin and copper alloy. In the recent era, Sn(II) is used in many fields in the industry such as aerospace, construction and home decor, electronics, jewelry manufacturing, telecommunications, paint/plastic industries, and agriculture via pesticides^1^. Sn(II) as fluoride is present in several dental care products such as toothpaste and mouth rinse^2^. Sn^2+^ has been utilized in dentistry as a chemical adjunct to prevent dental caries since 1950^3,4^. It was found to effectively inhibit Streptococcus mutants, which leads to tooth decay in human interproximal dental plaque and oral disease^5^. However, the wide utilization of toothpaste in our everyday life may increase the consumption of Sn^2+^ in the human body. The continuous use of Sn(II) becomes detrimental to our health and environment as well. The human can intake Sn(II) by breathing, through the skin, or by the consumption of food^6^. The accumulation of Sn(II) can induce acute and long-term effects in the human body such as eye irritations, heavy sweating, urination complications. Excess consumption of Sn(II) also finds severe immunotoxic and neurotoxic effects in humans causing symptoms such as disorders in the immune system, damage in liver functioning, chromosomal destruction, damage in the brain, and lack of red blood cells^7–10^. Recent studies revealed that the presence of an excess of Sn(II) in humans can easily be coordinated by white blood cells and enter into the cells by calcium channels, inducing DNA damage^11,12^. According to the guiding principles of the World Health Organization’s (WHO) for metals, the permissible limit of Sn(II) in drinking water and canned foods are 8.4 × 10^−4^–8.4 × 10^−3^ M and 2.105 × 10^−6^ M, respectively^13^. Due to the hazardous impacts of Sn(II) on humans, consumption of Sn(II) must be closely monitored.

The development of a highly sensitive expedient and readily accessible tool is the greatest requirement. However, various methods have already been performed to detect Sn(II)^14–20^, fluorimetric and colorimetric sensing method is one of the simplest and convenient sensing methods over other established detection techniques because it offers the advantage of ‘on spot’ real-time detection with naked eyes, low cost, portable, and wide applicability^21–29^. Hence, inspired with the requirement of active colorimetric probes, herein, we report the synthesis and sensing behavior of a novel colorimetric probe carbazole-naphthaldehyde conjugate (CNP) that exhibits high selectivity and sensitivity toward Sn^2+^ in the neutral aqueous medium (10 mM phosphate buffer, pH 7.0). The structure of the synthesized probe CNP was confirmed by detailed NMR (^1^H NMR, ^13^C NMR), HRMS, and X-ray analysis (Figure S1–S4, ESI†) as well as optimized by density functional theory. Significantly, the discriminative detection and quantification of Sn^2+^ in different toothpaste samples has also been accomplished using our synthesized probe CNP. To the best of our knowledge, this is the first colorimetric sensor displaying a distinguished recognition of Sn^2+^ in different toothpaste and mouth wash samples.

## Results and discussions

To explore the interaction pattern of the probe **CNP** with Sn^2+^, we have performed several experiments such as NMR titration, absorbance titration, pH titration, selectivity test and theoretical calculations.

### Crystallographic analysis

**Figure 1:**
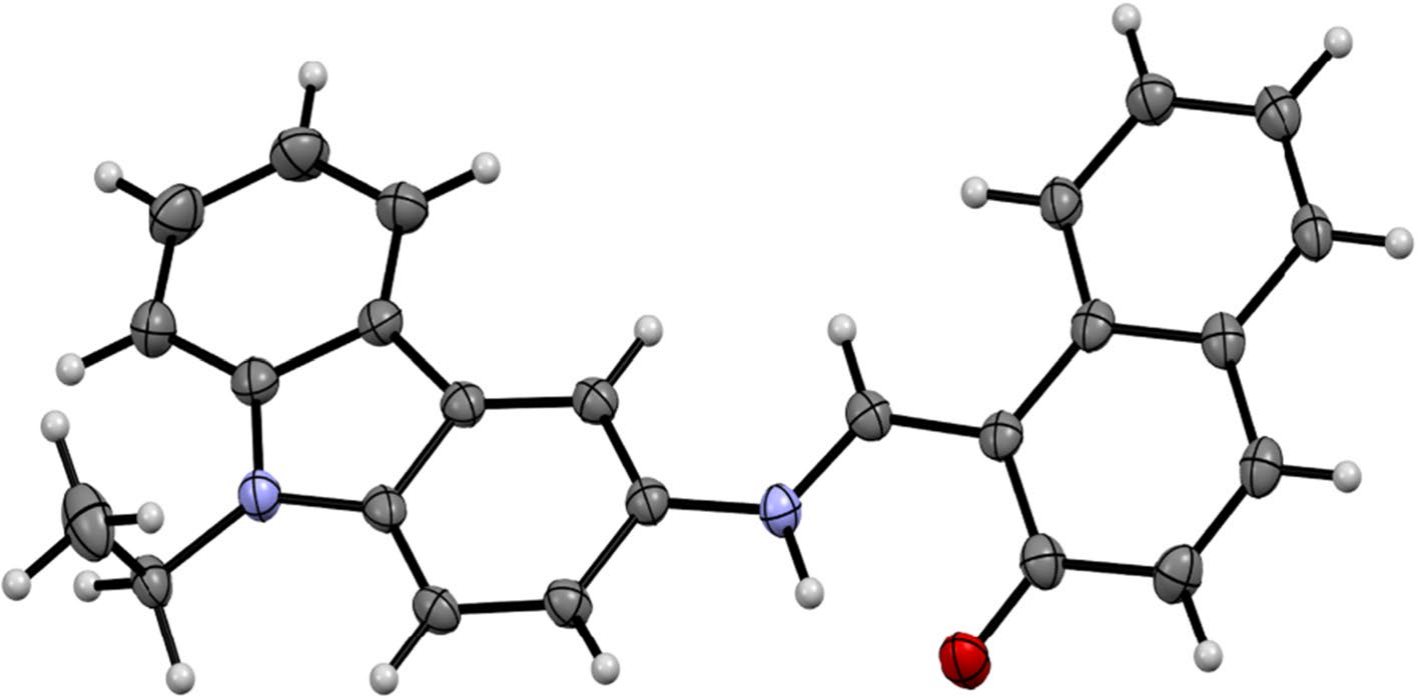
Displacement ellipsoid plot of **CNP**. Displacement ellipsoids are drawn at the 50% probability level.

Single crystals suitable for X-ray diffraction were obtained by slow evaporation of an acetonitrile solution of **CNP** at 3 °C. It crystallises in the monoclinic space group *P*2_1_/*n* (Fig. 1, Figure S4 in ESI†). Different than in solution where **CNP** is unequivocally present as the enol-imine tautomer (Figures S1, S2 in ESI†), the X-ray structure of **CNP** revealed that the compound is in the keto-enamine form in solid-state.

### NMR titration analysis

**Figure 2:**
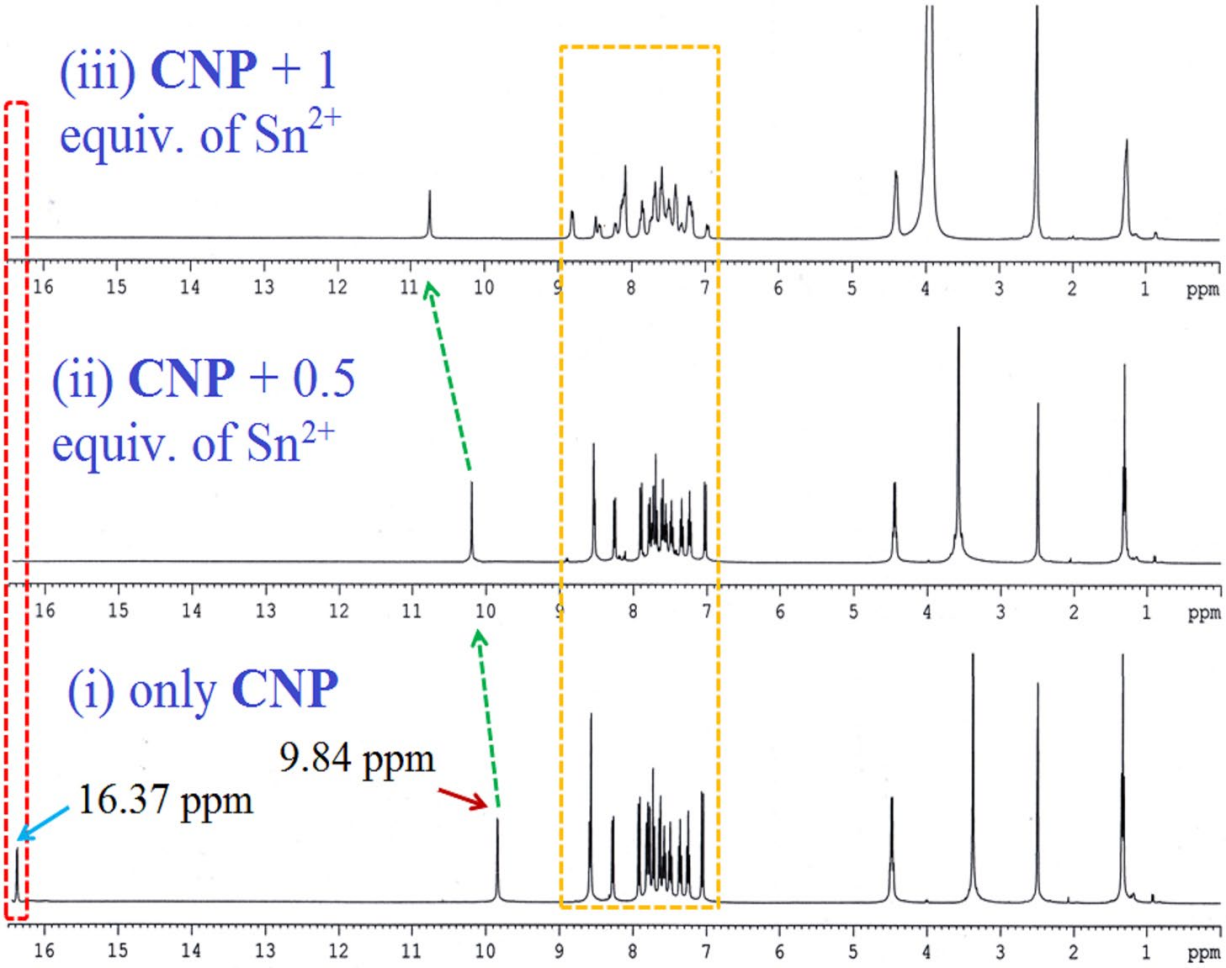
^1^H NMR titration [400 MHz] of **CNP** in DMSO-*d*_*6*_ at 25 °C and the corresponding changes after the addition of **Sn**^**2+**^ (SnCl_2_·2H_2_O) in D_2_O from (1) only **CNP**, (2) **CNP** + 0.5 equivalent of Sn^2+^, and (3) **CNP** + 1 equivalent of Sn^2+^.

Interactive properties of the probe **CNP** towards Sn^2+^ was investigated through ^1^H, ^13^C NMR titrations in DMSO-*d*_*6*_ and D_2_O. In ^1^H NMR titration, the aromatic –OH proton peak at 16.37 ppm abruptly disappears after addition of one equivalent of Sn^2+^ (SnCl_2_ dihydrate), followed by downfield shift of imine proton peak at 9.84 ppm. This phenomena indicates the formation of strong coordination between –OH, –N groups of **CNP** and Sn^2+^ (Fig. 2).

**Figure 3:**
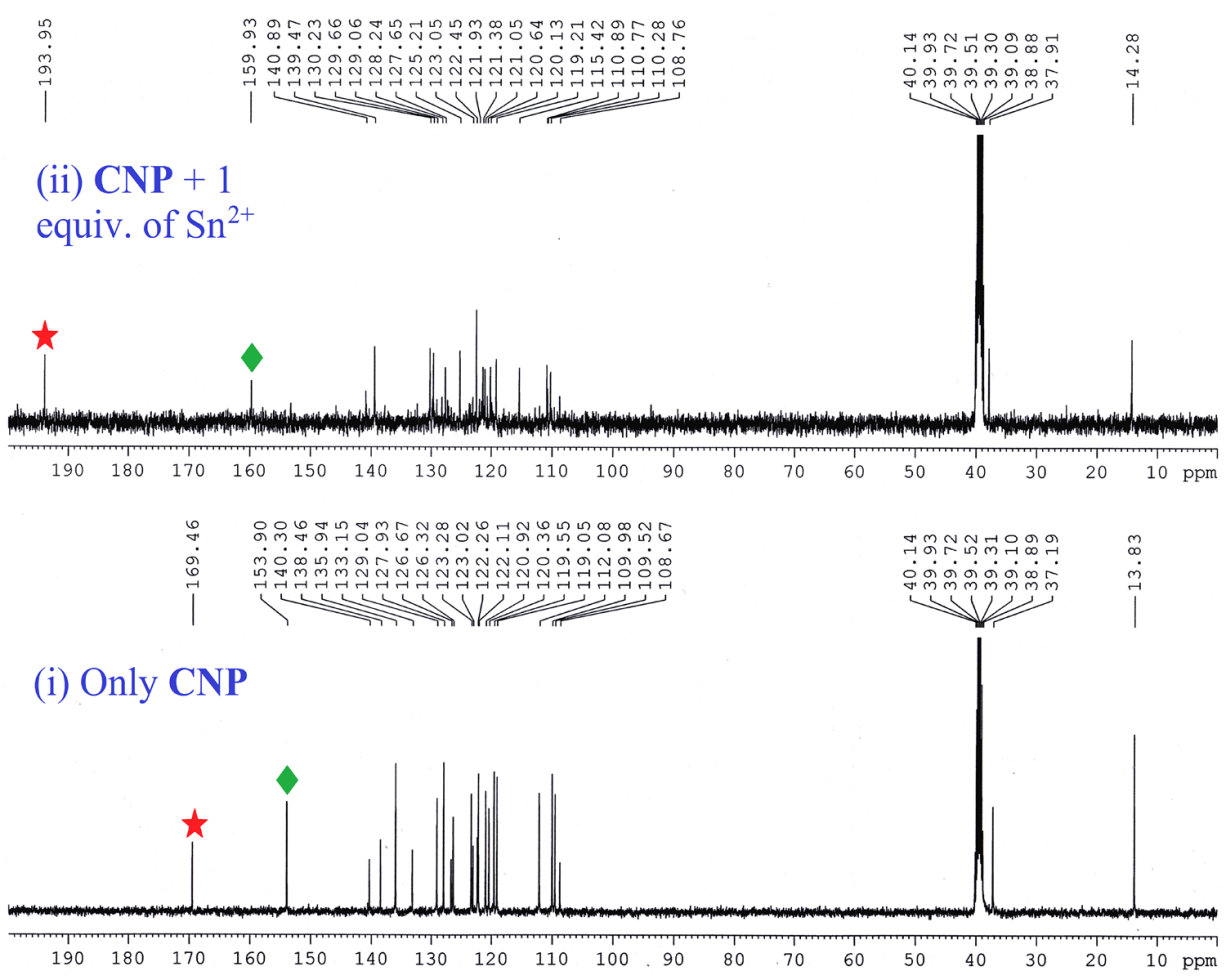
^13^C NMR titration [100 MHz] of **CNP** in DMSO-*d*_*6*_ at 25 °C and the corresponding changes after addition of Sn^2+^in D_2_O where (1) only **CNP**, and (2)**CNP + **1 equivalent of Sn^2+^.

Moreover in ^13^C NMR titration with the addition of one equivalent Sn^2+^, the carbon attached with –OH group shifts downfield from 169 to 193 ppm and also the imine ‘C’ peak at 153 ppm shifted towards 159 ppm with decreased intensity (Fig. 3). The above features also confirm the formation of **CNP–Sn**^**2+**^ complex.

### UV–Vis spectral behaviour of CNP with Sn^2+^

**Figure 4:**
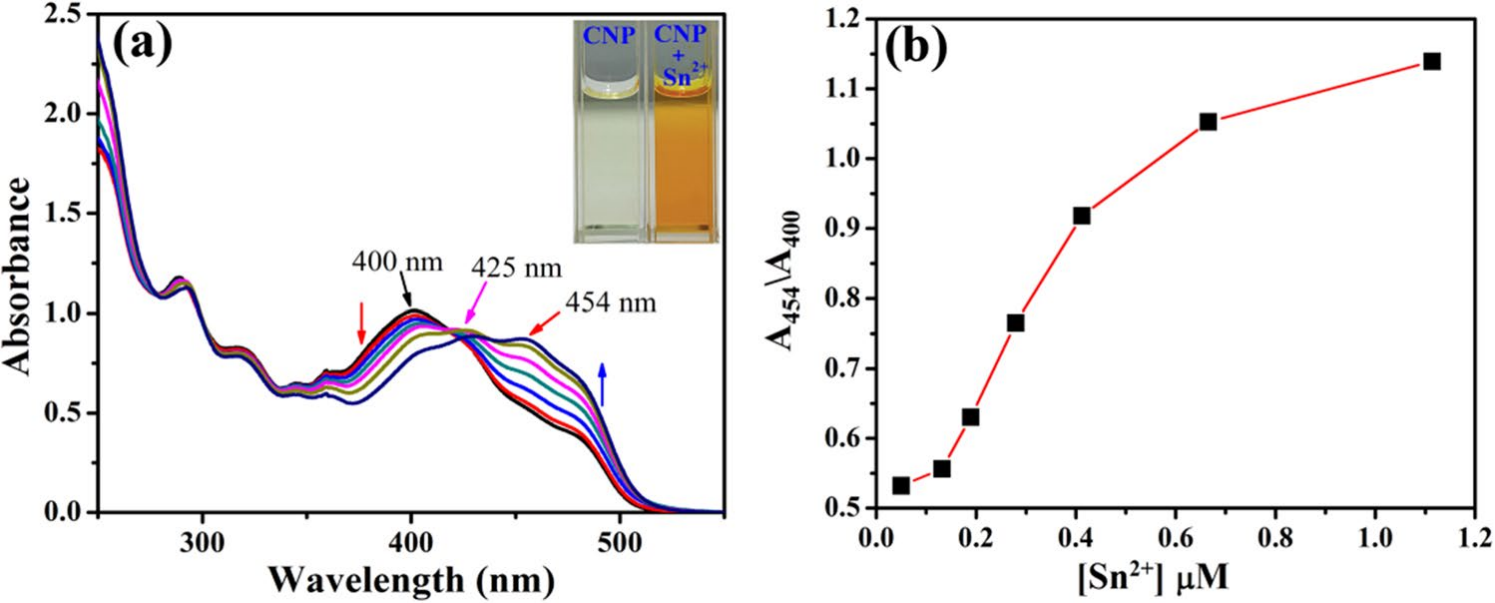
(**a**) UV–Vis absorption spectra of **CNP** (1 μM) upon incremental addition of Sn^2+^ up to 1.2 µM in CH_3_CN:H_2_O (1:8, v/v) at pH 7.0 (10 mM phosphate buffer) [inset: naked eye colour change of **CNP** on addition of Sn^2+^]. (**b**) Ratiometric change in absorbance with increasing concentration of Sn^2+^ at neutral pH.

The interactions between the probe **CNP** and Sn^2+^ was demonstrated by absorbance titration in acetonitrile:water (1:8, v/v) at neutral pH value (pH 7.0, 10 mM phosphate buffer). In the UV–Vis absorption spectra, we get a characteristic absorbance peak of **CNP** at 400 nm which decreases upon incremental addition of Sn^2+^, with an enhancement at 454 nm followed by a rapid colour change from pale yellow to deep orange. Furthermore, a notable isosbestic point at 425 nm indicates possible stronger interaction between **CNP** and Sn^2+^ (Fig. 4a). Ratiometric changes in absorbance with increasing concentration of Sn^2+^ have been represented in Fig. 4b.

Binding interactions determine 1:1 stoichiometric ratio of **CNP** and Sn^2+^ (Figure S5, ESI†) with a high association constant of 0.35 × 10^6^ M^-1^ from absorption spectra^30,31^ (Figure S6, ESI†). The detection limit of **CNP** for Sn^2+^ has been evaluated 0.85 nM (Figure S7, ESI†).

### Effect of pH value

pH titration clearly reflects that **CNP** is slight sensitive towards acidic pH values whereas the **CNP–Sn**^**2+**^ complex is pH independent (Figure S8, ESI†). So, we carried out all the experiments in the pH value range of 7.0 using 10 mM phosphate buffer.

### Colorimetric responses of CNP toward various metal ions

The colorimetric behaviour of the sensor probe **CNP** was evaluated upon the addition of various metal ions in the aqueous medium (10 mM phosphate buffer, pH 7.0). As depicted in Figure S9, the pale yellow color of the probe turned to deep orange with the addition of Sn^2+^. The synthesized probe **CNP** did not show any notable color changes with the addition of other metal ions. The specific color change of **CNP** with Sn^2+^ was attributed to several electron transitions in the **CNP–Sn**^**2+**^ complex such as π–π*, d–d, ligand–metal charge transfer, and metal–ligand charge-transfer effects. Furthermore, the comparative spectrophotometric response of **CNP** was also studied with these metal ions which confirms that our probe **CNP** selectivity sense Sn^2+^ over other metal ions (Figure S10, ESI†). Therefore, these experimental results indicate that our synthesized probe **CNP** shows remarkable selectivity and sensitivity towards Sn^2+^ over other analytes which could be a beneficial tool for practical approach.

### Theoretical calculations

Theoretical calculations were executed for **CNP** and **CNP–Sn**^**2+**^ complex systems using quantum chemical calculations at the DFT level LANL2DZ/6-31G** method basis set implemented at Gaussian 09 program^32^ and CPCM (Conductor like Polarizable Continuum Model) solvent model was used for solvent (water) effect incorporation. In the optimized structure of **CNP**, the positions of carbazole and naphthaldehyde units were nearly in the same plane. The optimized structure of the **CNP–Sn**^**2+**^ complex showed the formation of coordination bonds of Sn^2+^ with –OH and –N groups of **CNP**, which enhanced the stability of the complex (Figure S11, Table S1, ESI†).

**Figure 5:**
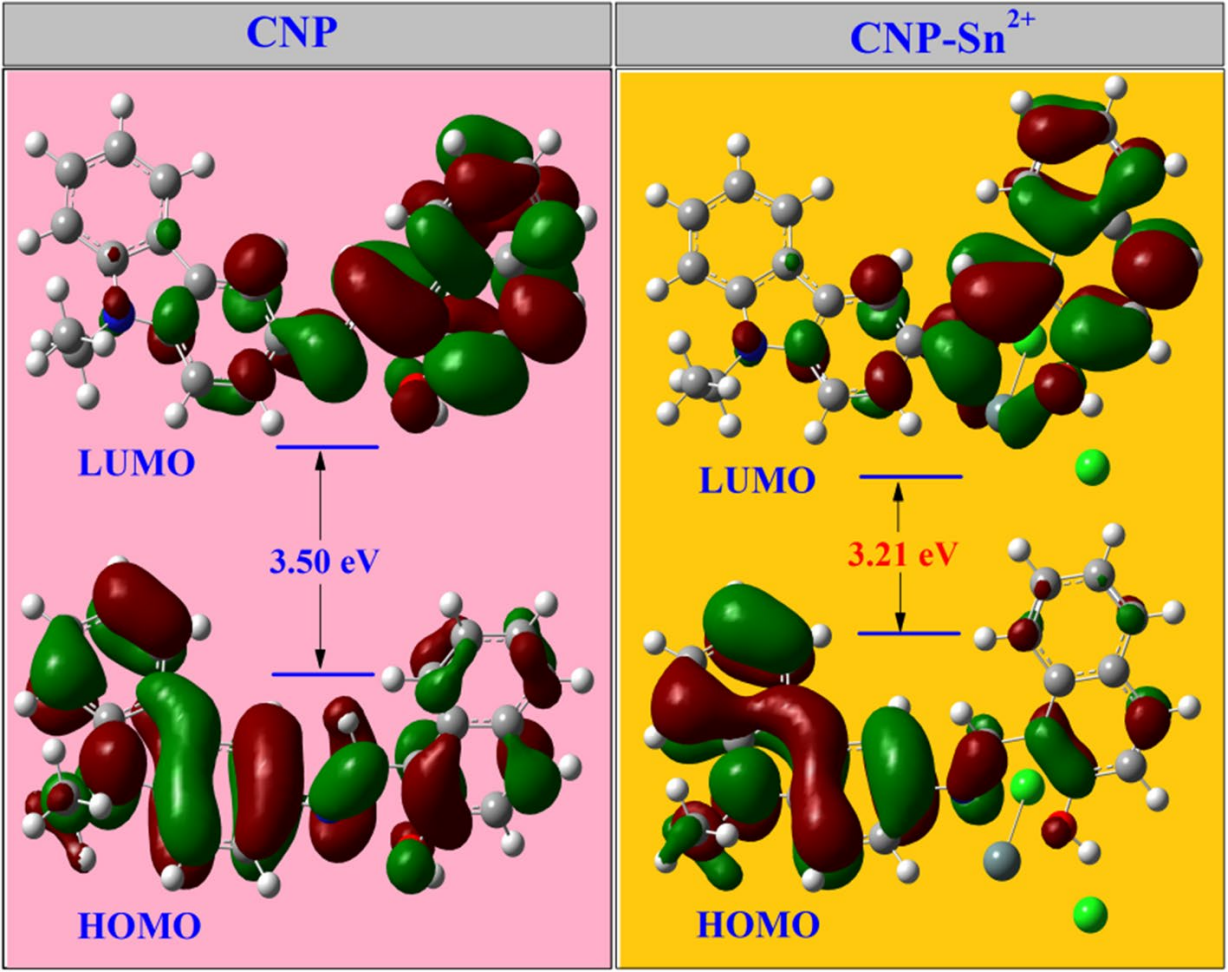
HOMO and LUMO distributions of **CNP** and **CNP–Sn**^**2+**^.

From TDDFT calculation, we can see that there is a sharp S_0_–S_1_ transition in **CNP** at 410 nm (oscillator strength f = 0.5188) which is very close to that experimentally observed value at 400 nm, responsible for the absorption of the carbazole moiety. Moreover, in **CNP–Sn**^**2+**^, the transition at 448 nm (S_0_–S_1_, f = 0.3563) indicates the π–π* electronic transition from the carbazole to naphthaldehyde moiety which is distinctly executed in the absorbance graph at 454 nm (Table S2, ESI†). Next, the energy distributions of HOMO and LUMO for **CNP** and its Sn^2+^ complex were examined (Table S3, ESI†). As shown in Fig. 5, the energy of the HOMO and LUMO orbital levels for the **CNP–Sn**^**2+**^ complex is lower than that of the probe **CNP**. Also, the HOMO–LUMO energy gap of **CNP** and **CNP–Sn**^**2+**^ complex was calculated with an energy difference of 0.29 eV.

**Figure 6:**
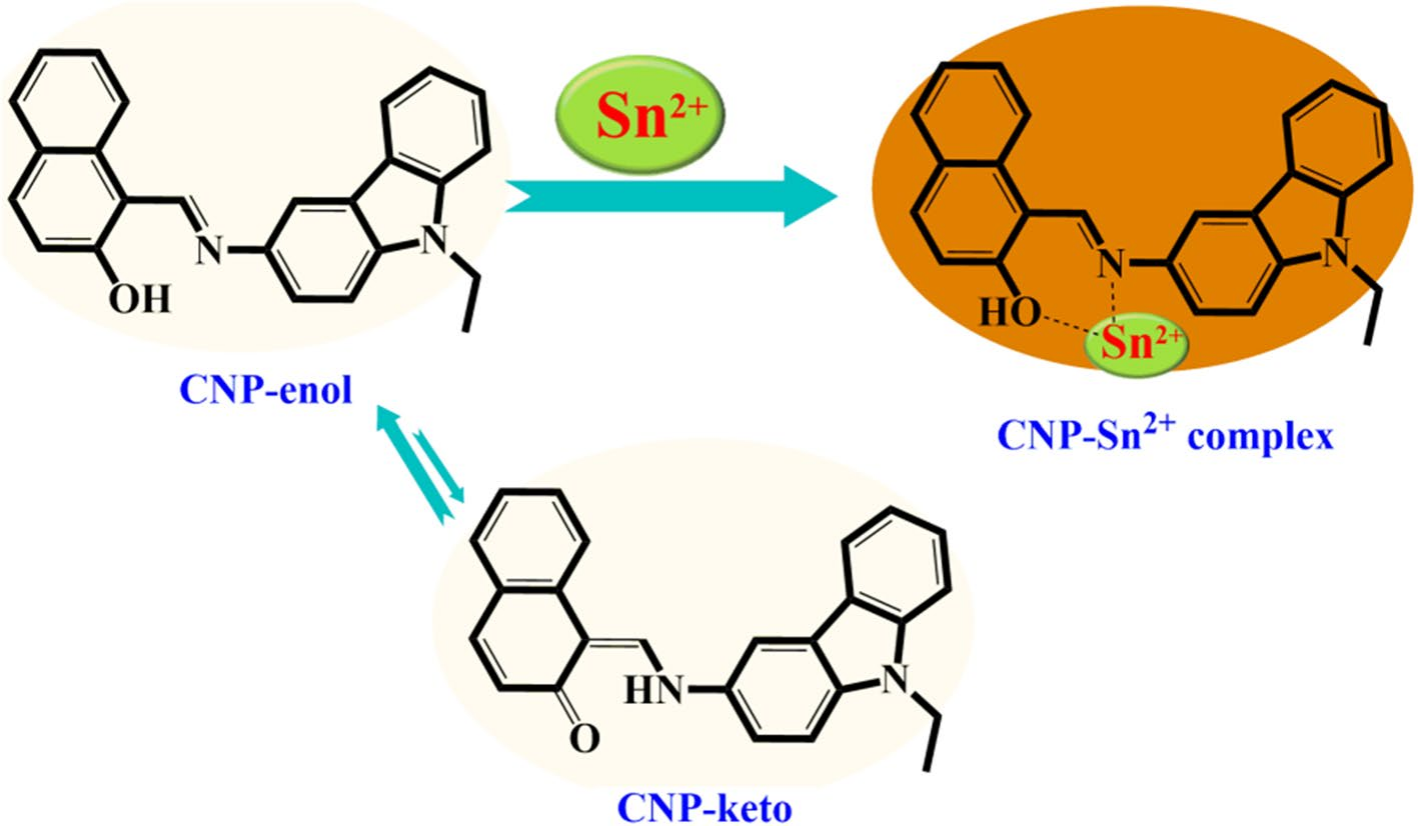
Proposed colorimetric detection mechanism of the **CNP–Sn**^**2+**^ complex.

These outcomes indicate that the effective resonance attraction obtained in the **CNP–Sn**^**2+**^ complex. The density of the orbital coefficient migrates from the carbazole unit to naphthaldehyde units in **CNP**, whereas in the **CNP–Sn**^**2+**^ complex, the orbital coefficient of total framework is moving towards Sn^2+^ via N–Sn^2+^ coordination bond. Hence, the obtained results imply the formation of a stable complex, which is consistent with the proposed binding mechanism. Furthermore, the mass of the **CNP–Sn**^**2+**^ complex has been checked which truly validated the binding mechanism (Figure S12, ESI†). The anticipated coordination mechanism of the **CNP–Sn**^**2+**^ complex is given in Fig. 6. In ^1^H NMR spectrum, the resonances assigned to the hydroxyl and imine groups are 16.37 ppm and 9.84 ppm, respectively. The naphthalene CO and imine C resonances in ^13^C NMR spectroscopy are observed at 169.46 and 153.90 ppm, respectively.

### Plausible mechanism and explanation

The naphthalene C–O bond length (1.282(3) Å) observed in the X-ray structure is characteristic of ketones rather than phenols, and the C–N bond (1.324(3) Å) is elongated relative to that of a typical imine^33,34^. Accordingly, CNP-enol is more susceptible to bind with Sn^2+^ through stronger hydrogen bonding which is also corroborated with IR experiments(Figure S13, ESI†).In consequence, the pale yellow color of **CNP** becomes the deep orange color due to the non-covalent interactions of **CNP** with Sn^2+^ (Fig. 6).

### Quantitative analysis

**Figure 7:**
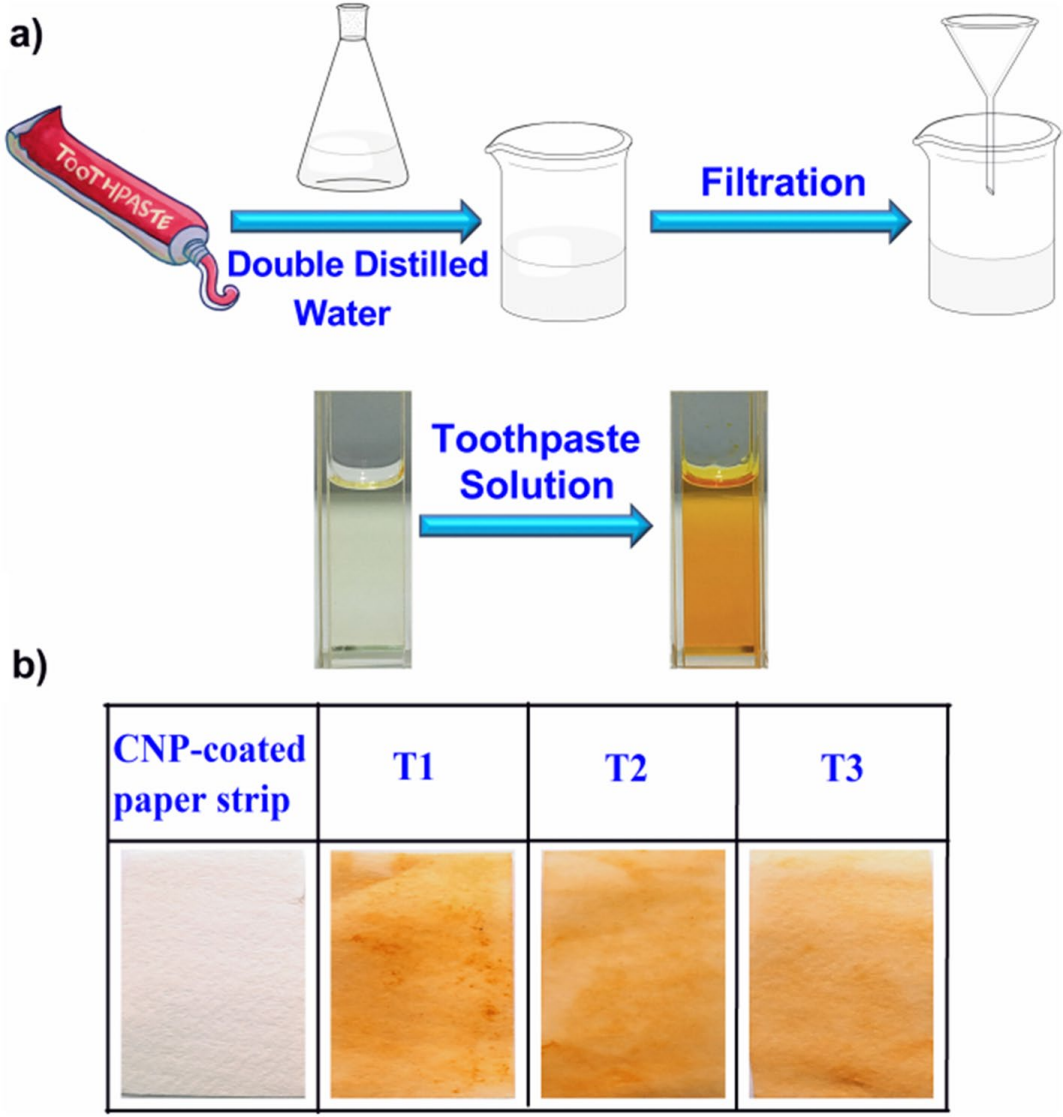
(**a**) Schematic diagram for estimation of Sn^2+^ in toothpaste samples using the probe CNP. (**b**) Display of naked-eye color change of CNP-coated paper strip after dipping in T1, T2, T3 solutions, respectively) (all experiments performed at pH 7.0, 10 mM phosphate buffer).

The excellent photophysical properties of the probe **CNP** toward Sn^2+^, such as high sensitivity and selectivity at physiological pH encouraged us to further evaluate the potential of the probe for realistic approach. The specific and selective recognition of Sn^2+^ by the chemosensor **CNP** was also examined in three different toothpaste samples. In this work, we took commercially available toothpastes from three different brands (T1, T2, T3). The details procedure of the preparation of toothpaste solutions was described in ESI†. Toothpaste solutions were then added to the **CNP** solution, a rapid orange color change resulted after few minutes (Fig. 7a). Furthermore, the method was also applied to recognize Sn^2+^ by a simple paper strip in different toothpaste solutions. Three paper strips soaked in **CNP** were dipped separately in the different toothpaste solutions (T1, T2, T3), and Fig. 7b shows the respective color changes of **CNP**-coated paper strip after dipping in the toothpaste solutions T1, T2, T3 respectively.

**Figure 8:**
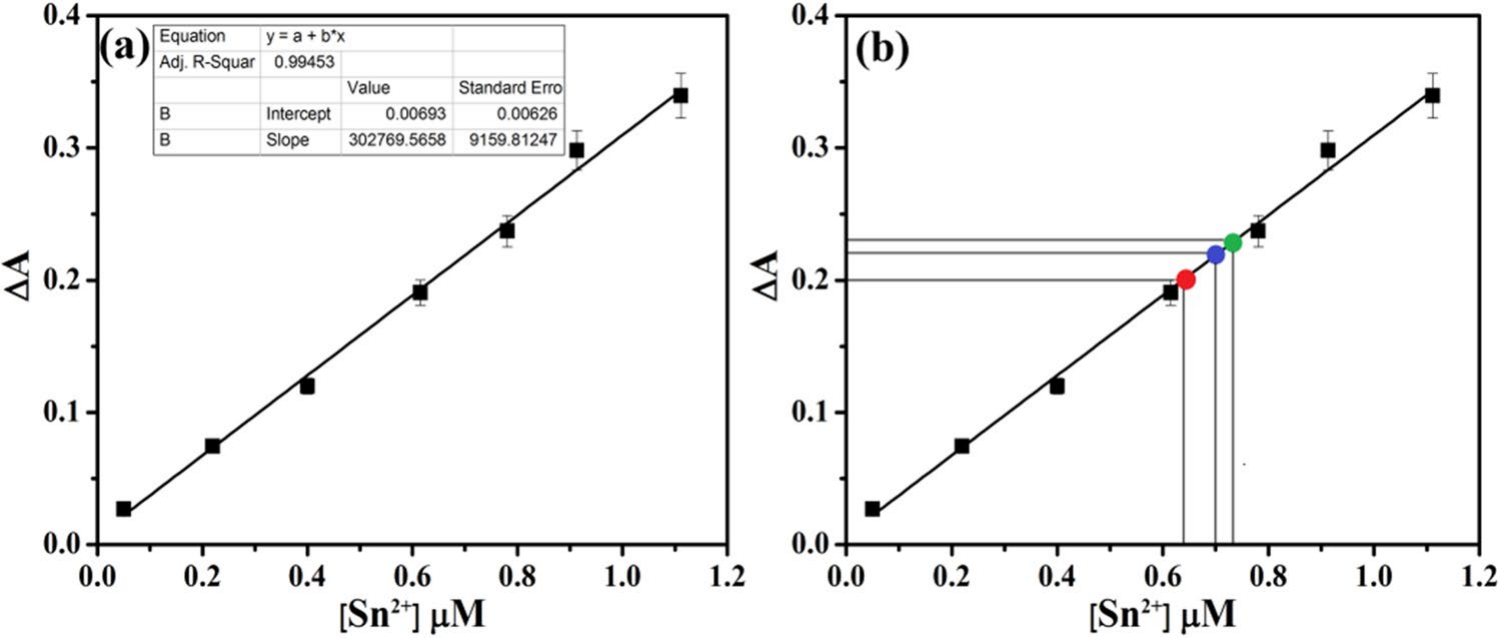
(**a**) Standard fluorescence curve obtained for the estimation of Sn^2+^ ions. (**b**) Estimation of unknown concentration of Sn^2+^ ions (red, blue and green point) in the different toothpaste samples from the standard fluorescence curve. Standard deviations are represented by error bar (n = 3).

The concentration of Sn^2+^ was also quantified from these three different toothpaste samples (T1, T2, T3). For this work, the above-mentioned toothpaste samples were subjected to colorimetric analysis at pH 7.0 (10 mM phosphate buffer) to quantify the amount of Sn^2+^ present therein. Sn^2+^ was quantified from these given samples by **CNP** (1 µM) by virtue of its selective and direct recognition properties. All estimations were done in triplicate. Concentrations of Sn^2+^ were estimated by comparison with the **CNP–Sn**^**2+**^ standard absorbance curve. From the standard curve it was found that the concentration of Sn^2+^ were 0.73 µM, 0.70 µM and 0.64 µM in 100 µL of T1, T2 and T3 samples, respectively (Fig. 8, Table 1). Concentration of Sn^2+^ was further quantified from two different mouth wash samples (M1, M2) using above mentioned procedure and the respective values are 0.25 µM and 0.28 µM in 100 µL sample solution (Table 2).

**Table 1:** Determination of [**Sn**^**2+**^] in algae solutions under UV-lamp.

Toothpaste sample	Conc. of CNP (µM)	Amount of toothpaste sample taken (µL)	Conc. of Sn^2+^ (µM)	Average conc. of Sn^2+^ (µM)
T1	1	100	0.73	0.73
		100	0.74	
		100	0.72	
T2	1	100	0.69	0.70
		100	0.70	
		100	0.71	
T3	1	100	0.63	0.64
		100	0.65	
		100	0.64

**Table 2:** Determination of [Sn^2+^] in different mouth wash samples.

Mouth wash sample	Conc. of CNP (µM)	Amount of Mouth wash sample taken (µL)	Conc. of Sn^2+^ (µM)	Average conc. of Sn^2+^ (µM)
M1	1	100	0.25	0.25
		100	0.23	
		100	0.24	
M2	1	100	0.29	0.28
		100	0.27	
		100	0.28	

## Conclusion

In conclusion, a new carbazole-naphthaldehyde based colorimetric probe **CNP** was successfully synthesized for selective recognition of Sn^2+^ in the aqueous medium under physiological pH value. The structure of the synthesized probe **CNP** was analyzed by single crystal X-ray diffraction which represents the presence of keto (CNP-keto) form in its solid state. The sensing mechanism has been triggered by the strong coordination bonding of CNP-enol with Sn^2+^, which was confirmed by absorbance, ^1^H and ^13^C NMR spectroscopy as well as mass spectrometry (HRMS). Theoretical calculations were also performed to justify the binding mechanism and optical behavior of the sensor probe. **CNP** showed high selectivity and sensitivity for Sn^2+^ even in the presence of other metal ions. The detection limit of the probe for Sn^2+^ was calculated to be 85 nM, which is much lower than the WHO permissible amount of Sn^2+^ in drinking water. We further demonstrated that **CNP** has been utilized as a colorimetric sensor to detect and quantify trace amounts of Sn^2+^ in different toothpaste and mouth wash samples. A handy and accessible paper strip method has been proposed for this purpose. Being a potential probe, it can be used as an expedient ‘in-field’ approach to estimate Sn^2+^ for environmental and industrial purposes for sustainable and environment-friendly industrial production.

## Experimental section

### Materials and methods

All the reagents were purchased from Sigma-Aldrich Pvt. Ltd. (India). Unless otherwise mentioned, materials were obtained from commercial suppliers and were used without further purification. Solvents were dried according to standard procedures. Elix Millipore water was used in all respective experiments. ^1^H and ^13^C NMR spectra were recorded on a Bruker 400 MHz instrument. For NMR spectra, DMSO-*d*_*6*_ and for NMR titration DMSO-*d*_*6*_ and D_2_O were used as solvent using TMS as an internal standard. Chemical shifts are expressed in δ ppm units and ^1^H–^1^H and ^1^H–^13^C coupling constants in Hz. The mass spectrum (HRMS) was carried out using a micromass Q-TOF Micro™ instrument by using methanol as a solvent. UV spectra were recorded on a SHIMADZU UV-3101PC spectrophotometer. FT-IR data were recorded on Shimadzu IRAffinity-1S Fourier transform infrared spectrometer (Spectrum Two) by ATR technique. The following abbreviations are used to describe spin multiplicities in ^1^H NMR spectra: s = singlet; d = doublet; t = triplet; m = multiplet. Single crystal X-ray data of **CNP** was measured using a dual-source Rigaku Super Nova diffractometer equipped with an Atlas detector and an Oxford Cryostream cooling system using mirror-monochromated Cu-K_α_ radiation (λ = 1.54184 Å). Data collection and reduction for both compounds were performed using the program *CrysAlisPro*^35^ and Gaussian face-index absorption correction method was applied^35^. The structures were solved with Direct Methods (*SHELXS*)^36–38^ and refined by full-matrix least-squares based on *F*^*2*^ using *SHELXL*-2015^36–38^. Non-hydrogen atoms were assigned anisotropic displacement parameters unless stated otherwise. The hydrogen atom bonded to nitrogen was located from Fourier difference maps and refined with an N–H distance restraint of approximately 0.96 Å. Otherhydrogen atoms were placed in idealised positions and included as riding. Isotropic displacement parameters for all H atoms were constrained to multiples of the equivalent displacement parameters of their parent atoms with U_iso_(H) = 1.2 U_eq_(parent atom). The single crystal X-ray data, experimental details as well as CCDC number are given in the Supporting Information.

### Synthetic procedure of CNP

**Figure 9:**
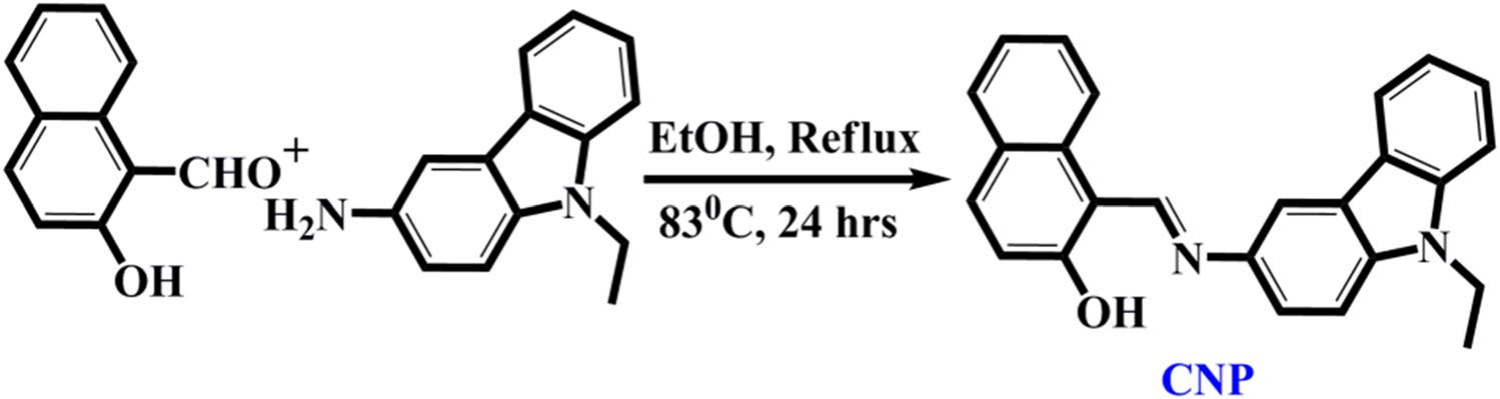
Synthesis of the probe **CNP**.

In a 100 mL round bottom flask, 2-hydroxy naphthaldehyde (1.0 g, 5.8 mmol) in 30 ml ethanol was vigorously stirred at ambient temperature for few minutes. Then, 3-amino-9-ethyl carbazole (1.46 g, 6.95 mmol) was dissolved in ethanol (10 mL) and added dropwise to the solution. The reaction mixture was refluxed for 24 h at 83 °C. After completion of the reaction (monitored by TLC), the solvent was evaporated completely under reduced vapor pressure, then extracted with chloroform and water. After drying it over anhydrous Na_2_SO_4_, the organic layer was evaporated completely to get the solid product. This product was purified by column chromatography with the eluent CHCl_3_:PET (5:1, v:v) to get the product **CNP** with 86% yield (Fig. 9). ^1^H NMR (400 MHz, DMSO-*d*_*6*_): δ (ppm) = 16.37 (s, 1H), 9.84 (s, 1H), 8.57–8.59 (d, 2H, J = 8 Hz), 8.26–8.28 (d, 1H, J = 8 Hz), 7.90–7.92 (d, 1H, J = 8 Hz), 7.77–7.82 (t, 2H, J = 20 Hz), 7.71–7.73 (d, 1H, J = 8 Hz), 7.62–7.64 (t, 1H, J = 8 Hz), 7.55–7.57 (t, 1H, J = 8 Hz), 7.47–7.51 (t, 1H, J = 16 Hz), 7.34–7.37 (t, 1H, J = 12 Hz), 7.23–7.26 (t, 1H, J = 12 Hz), 7.04–7.06 (d, 1H, J = 8 HZ), 4.47–4.49 (q, 2H, J = 8 Hz), 1.31–1.34 (t, 3H, J = 12 Hz). ^13^C NMR (100 MHz, DMSO-*d*_*6*_): δ (ppm) = 169.46, 153.90, 140.30, 138.46, 135.94, 133.15, 129.04, 127.93, 126.67, 126.32, 123.28, 123.02, 122.26, 122.11, 120.92, 120.36, 119.55, 119.05, 112.08, 109.98, 109.52, 108.67, 37.19, 13.83. HRMS (TOF MS): (m/z, %): Calcd. for C_25_H_20_N_2_O: 364.1576. Found: m/z = 365.1283 (M + H^+^).

## Supplementary Information

Supplementary Information.
