# Supplementary material for: A handy and accessible tool for identification of Sn(II) in toothpaste
Source: Sci Rep. 2022 Feb 10;12:2305. doi: 10.1038/s41598-022-06299-0 (PMC8831574; doi:10.1038/s41598-022-06299-0)
Supplement: Supplementary file 1 — Supplementary Information. [file 41598_2022_6299_MOESM1_ESM.docx]

**Supporting information**

**A handy and accessible tool for identification of Sn(II) in toothpaste**

Shampa Kundu,^a^ Khai-Nghi Truong,^b^ Shrabani Saha,^a^ Kari Rissanen^b^ and Prithidipa Sahoo*^a^

^a^Department of Chemistry, Visva-Bharati University, Santiniketan, 731235 West Bengal, India

^b^University of Jyvaskyla, Department of Chemistry,P.O. Box 35, Survontie 9 B, 40014 Jyväskylä, Finland

Email: prithidipa@hotmail.com

**Table of Content**

1. NMR Studies S2

2. Mass Spectrum of CNP S3

3. Crystallographic Data S4

4. UV-Vis Titration S4

5. Job’s Plot for Determining the Stoichiometry of Interaction by Absorbance Method S5

6. Calculation of Binding Constants of CNP Towards Sn^2+^ S6

7. Calculation of Limit of Detection (LOD) of CNP with Sn^2+^ S7

8. pH Titration Curve of CNP upon Addition of Sn^2+^ S7

9. Absorbance Comparative Studies of CNP with Different Metal Ions S8

10. Details of Energy Calculations Using Density Functional Theory (DFT) S9

11. Partial HRMS of the mixed assay system S10

12. IR spectroscopy S11

13. Preparation of Toothpaste Solution S11

14. References S12

**1. NMR Studies**

**^1^H NMR of CNP in DMSO-*d_6_*:**

**
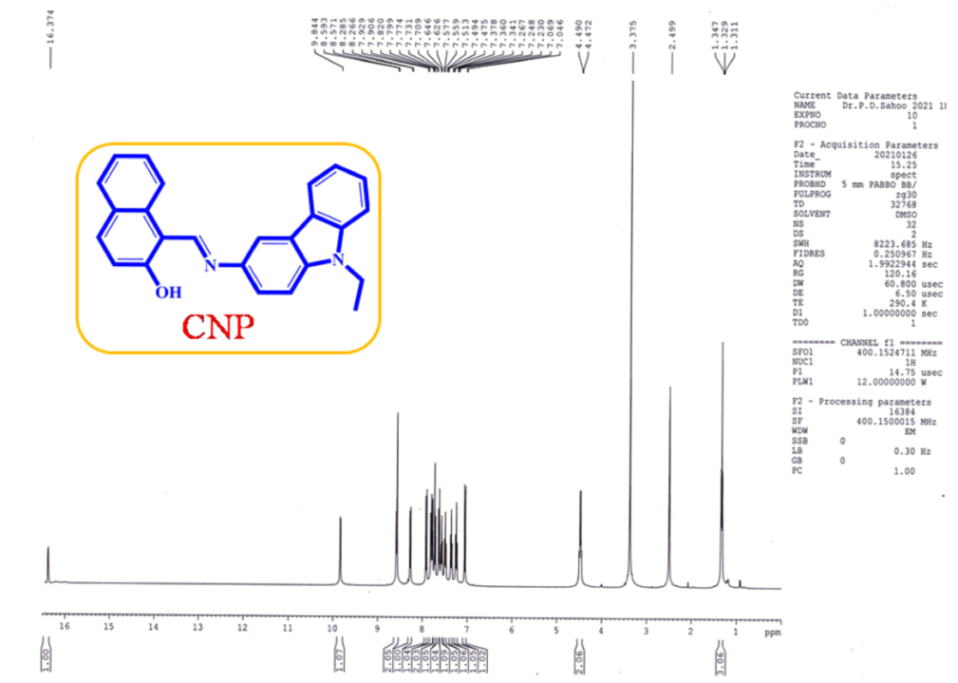
**

**Figure S1. ^1^**H NMR of **CNP** in DMSO-*d_6_* (400 MHz, 25°C).

**^13^C NMR of CNP in DMSO-*d_6_*:**

**
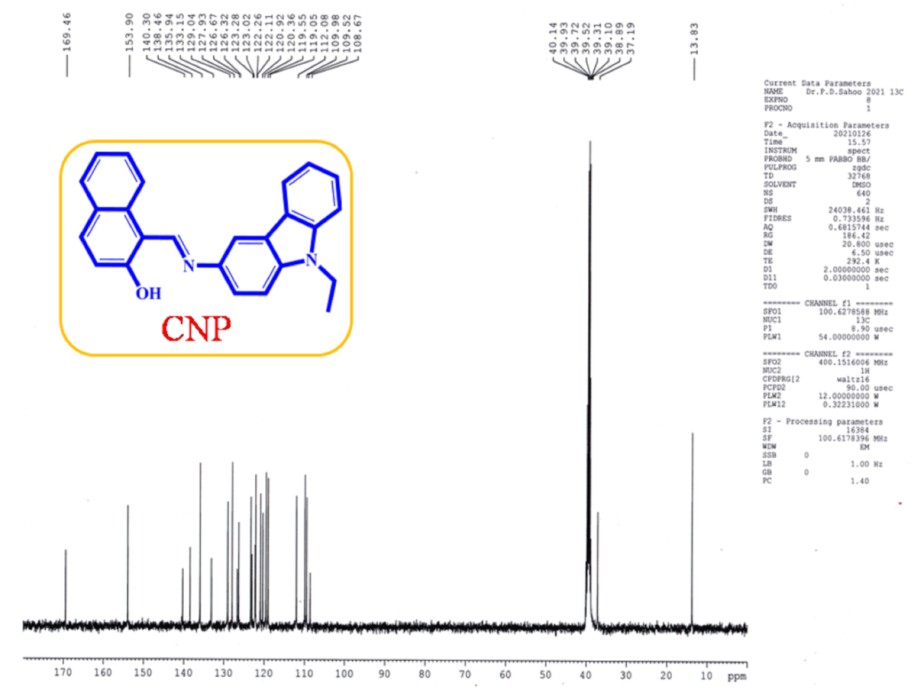
**

**Figure S2. ^13^**C NMR of **CNP** in DMSO-*d_6_* (100 MHz, 25°C)

**2. Mass Spectrum of CNP**

**
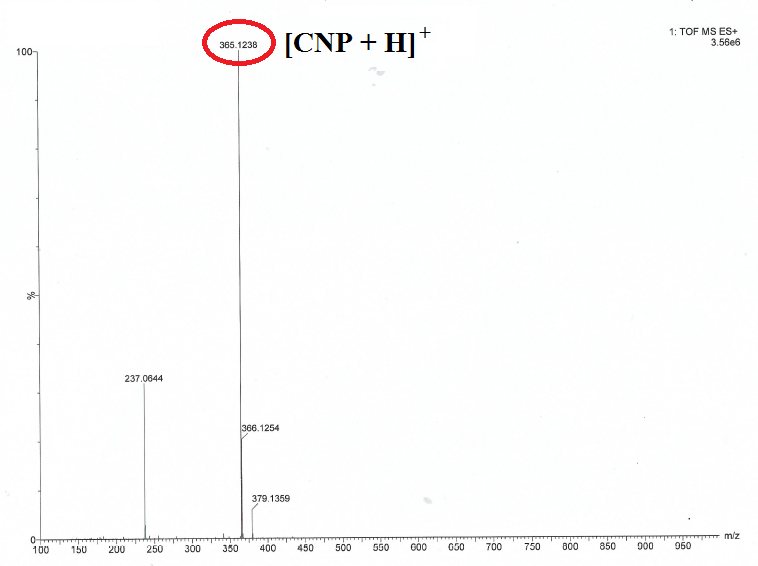
**

**Figure S3.** HRMS of **CNP**.

**3. Crystallographic Data**

Crystal data for **CNP** (obtained *via* slow evaporation from CH_3_CN at 3 °C): CCDC-2110993, C_25_H_20_N_2_O, M = 364.43 gmol^–1^, yellow rod, 0.17 × 0.04 × 0.04 mm^3^, monoclinic, space group *P*2_1_/*n*(No. 14), a = 16.2442(16) Å, b = 6.0442(5) Å, c =18.9730(18) Å, α = 90°, β = 94.569(3)°, γ = 90°, V = 1856.9(3) Å^3^, Z = 4, D_calc_ = 1.304 gcm^–3^, F(000) = 768, µ = 0.627 mm^–1^, T = 120(2) K, θ_max_ = 73.29°, 13456 total reflections, 2148 with I_o_> 2σ(I_o_), R_int_ = 0.0920, 3513 data, 254 parameters, no restraints, GooF = 1.011, R_1_ = 0.0550 and wR_2_ = 0.1179 [I_o_> 2σ(I_o_)], R_1_ = 0.1029 and wR_2_ = 0.1407 (all reflections), 0.264< d∆ρ <-0.258 eÅ^–3^.

**
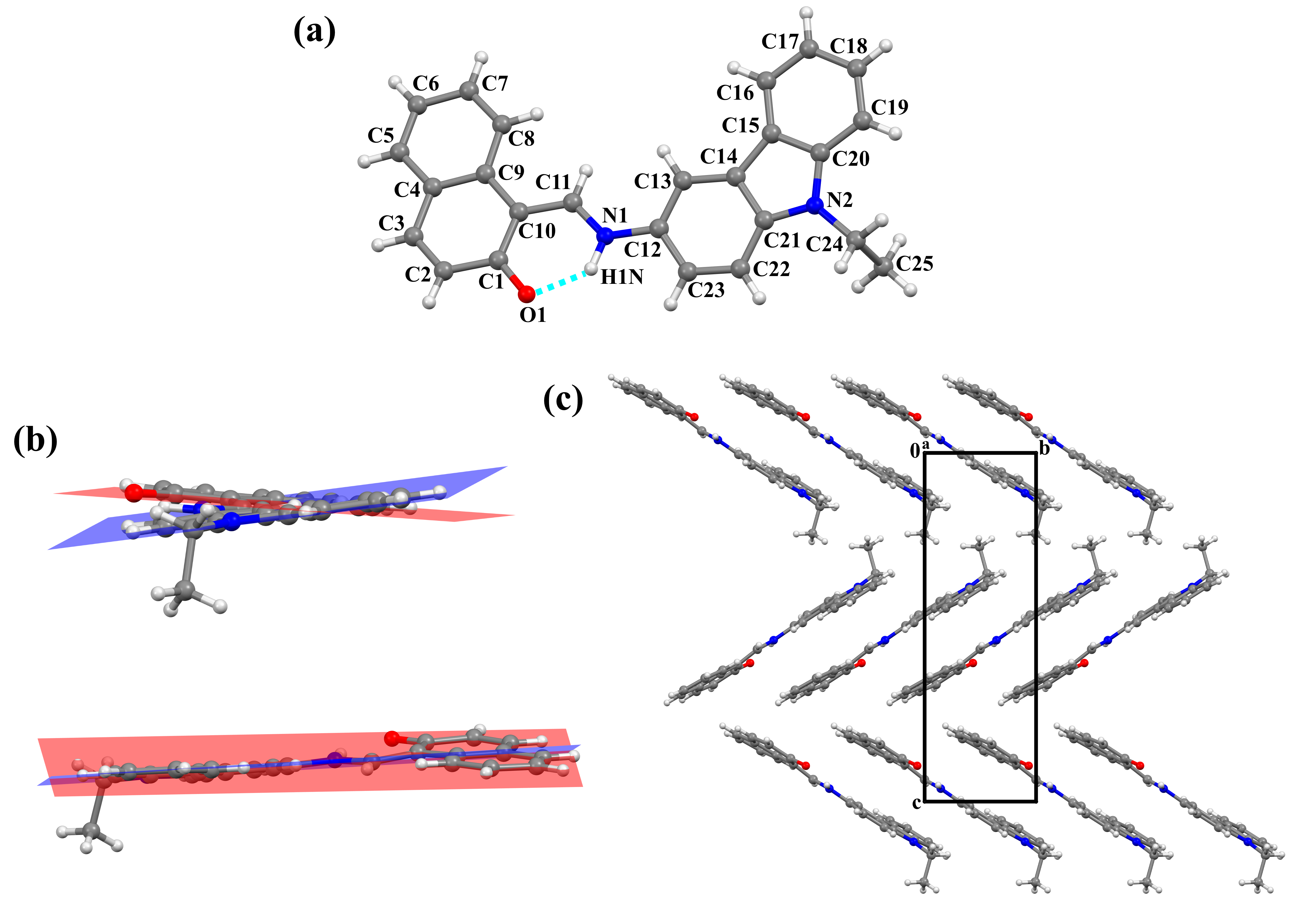
**

**Figure S4.** (a) Ball-and-stick model of **CNP** (with atom numbering); (b) different views of **CNP** displaying the deviation from neighbouring heterocyclic ring mean planes using the programme MERCURY^[1]^; and (c) crystal packing of **CNP** showing π^…^π stacking of neighboring molecules. Selected bond distances (Å) and angle (°): O1−C1 1.282(3), C1−C10 1.427(4), C10−C11 1.407(4), C11−N1 1.324(3), N1−H1N 0.9600, O1^…^N1 2.550(3), O1^…^H1N−N1 134.04(9).

**4. UV-Vis Titration**

A stock solution of **CNP** (1 μM) was prepared in acetonitrile:water (1:8, v/v). Sn^2+^ solutions of different concentrations were prepared in Millipore water. All experiments were carried out in acetonitrile:water (1:8, v/v), buffered with 10 mM phosphate buffer (pH 7.0). During the titration, each time 1 μM solution of **CNP** was filled in quartz optical cell of 1 cm optical path length and Sn^2+^ solution was added into the quartz optical cell gradually using a micropipette until saturation. Spectral data were recorded immediately after the addition of Sn^2+^solution.

**5. Job’s Plot for Determining the Stoichiometry of Interaction by Absorbance Method**

**
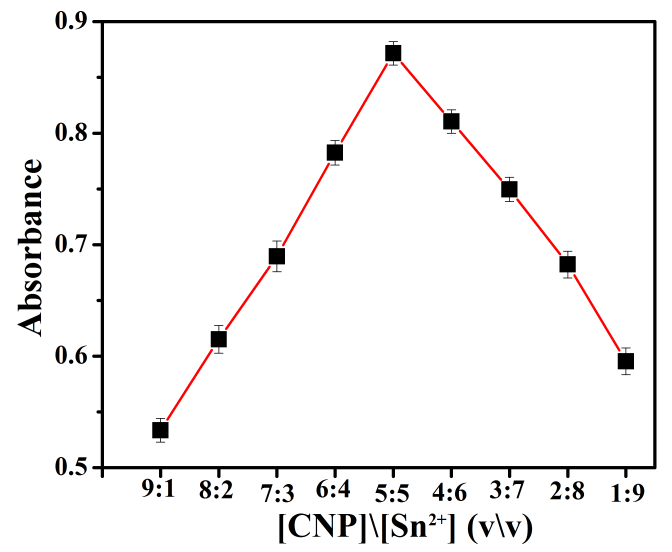
**

**Figure S5.** Job’s plot of **CNP** (c =1µM) with Sn^2+^ (1 µM) in acetonitrile: water (1:8, v/v) at neutral pH value (pH 7.0, 10 mM phosphate buffer) by absorbance method, which indicate 1:1 stoichiometry for **CNP** with Sn^2+^.Standard deviations are represented by error bar (n=3).

**6. Calculation of Binding Constants of CNP towards Sn^2+^**

**
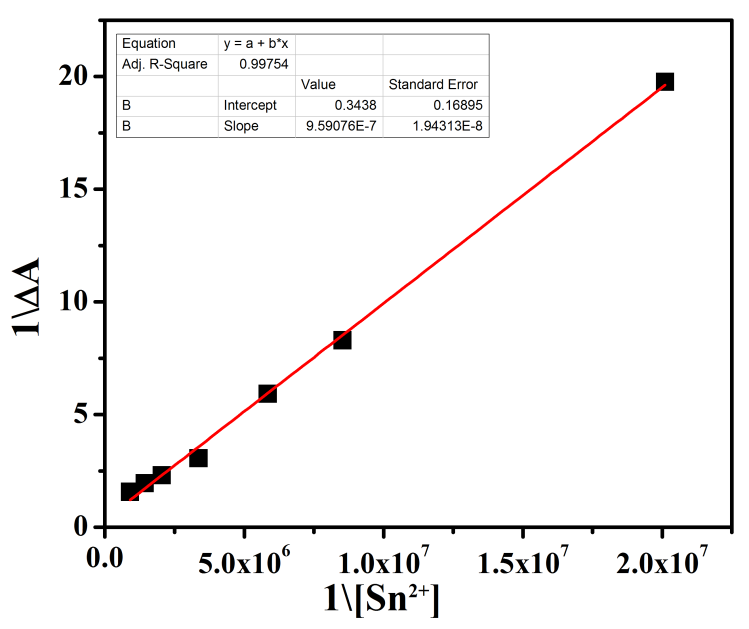
**

**Figure S6.** Linear regression analysis for the calculation of binding constant values of **CNP** towards Sn^2+^.

The association constant (K_a_) of **CNP** for sensing Sn^2+^ was determined from the equation: K_a_ = intercept/slope. From the linear fit graph we get intercept= 0. 3438, and slope = 9.59076×10^-7^.Thus we get, **K_a_** = (0.3438) / (9.59076×10^-7^) = **0.35 × 10^6^ M^-1^**.

**7. Calculation of Limit of Detection (LOD) of CNP with Sn^2+^**

The detection limit of the chemosensor **CNP** for Sn^2+^was calculated on the basis of UV-Vis titration. The limit of detection (LOD) of **CNP** for sensing Sn^2+^ was determined from the following equation^[2,3]^:

LOD = K × SD/S

where K = 2 or 3 (we take 3 in this case); SD is the standard deviation of the blank receptor solution; S is the slope of the calibration curve.


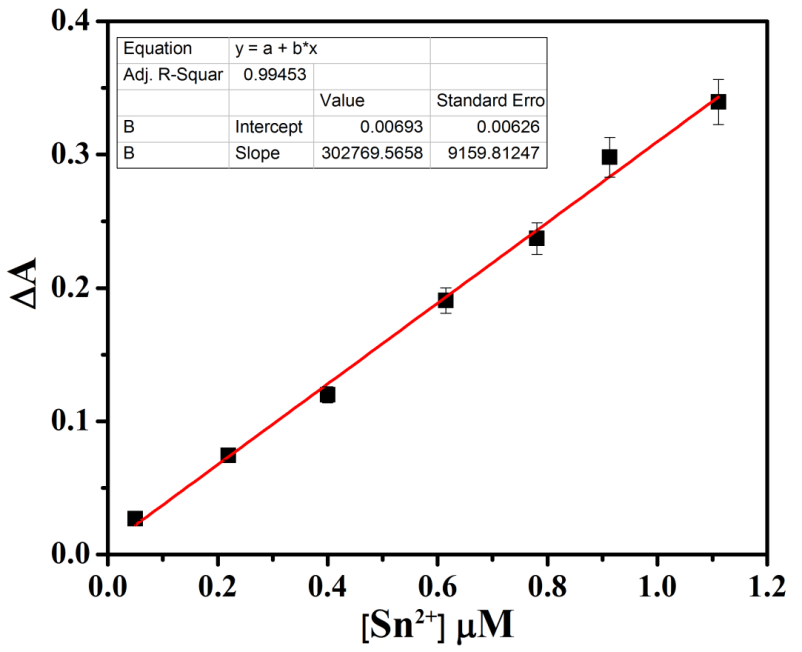


**Figure S7.** Linear fit curve of **CNP** at 454 nm with respect to Sn^2+^concentration. Standard deviations are represented by error bar (n=3).

From the linear fit graph, we get slope = 302769.5658, and SD value is 0.00858.Thus using the above formula we get the Limit of Detection = 8.5×10^-8^ M, i.e.85 nM. Therefore **CNP** can detect Sn^2+^up to this very lower concentration by colorimetric technique.

**8. pH Titration Curve of CNP upon Addition of Sn^2+^**


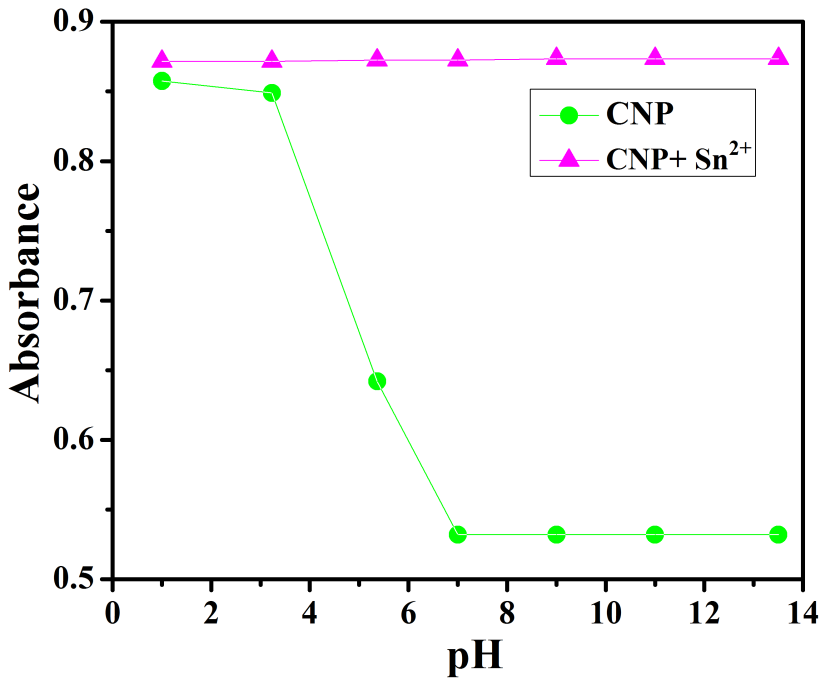


**Figure S8.** Effect of pH value on the absorbance of **CNP**(1 μM) in the absence of Sn^2+^ (green line) and in the presence of Sn^2+^ (10 μM, pink line).

**9. Absorbance Comparative Studies of CNP with Different Metal Ions**

**
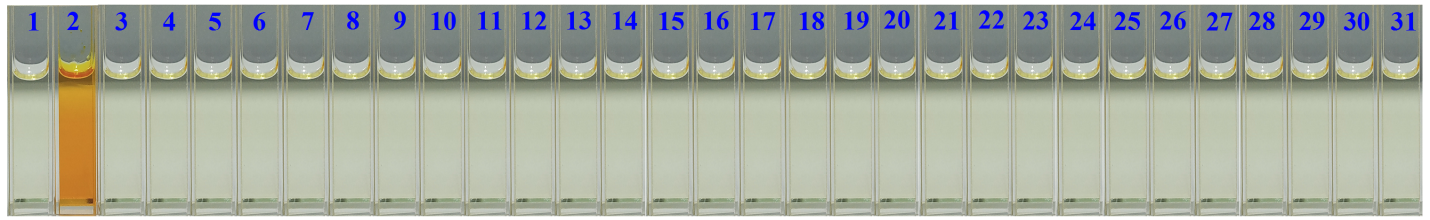
**

**Figure S9.** Photograph of colorimetric changes of **CNP** (1µM) to various metal ions (10 µM) [From left to right: 1) Only **CNP**; **CNP** with 2) Sn^2+^, 3) Sn^4+^, 4) Cd^2+^, 5) Pb^2+^, 6) Hg^2+^, 7) Mg^2+^, 8) Mn^2+^, 9) Ca^2+^, 10) Ba^2+^, 11) Sr^2+^, 12) Co^2+^, 13) Ni^2+^, 14) Pt^2+^, 15) Pd^2+^, 16) Pd^0^, 17) Fe^2+^, 18) Cu^2+^, 19) 20) Zn^2+^, 21) Fe^3+^, 22) Al^3+^, 23) Cr^3+^, 24) Ru^3+^, 25) Au^3+^, 26) Zr^4+^, 27) Hf^4+^, 28) Li^+^,29) Na^+^, 30) K^+^, 31) Ag^+^ in acetonitrile:water (1:8, v/v, pH 7.0, 10 mM phosphate buffer) solution].


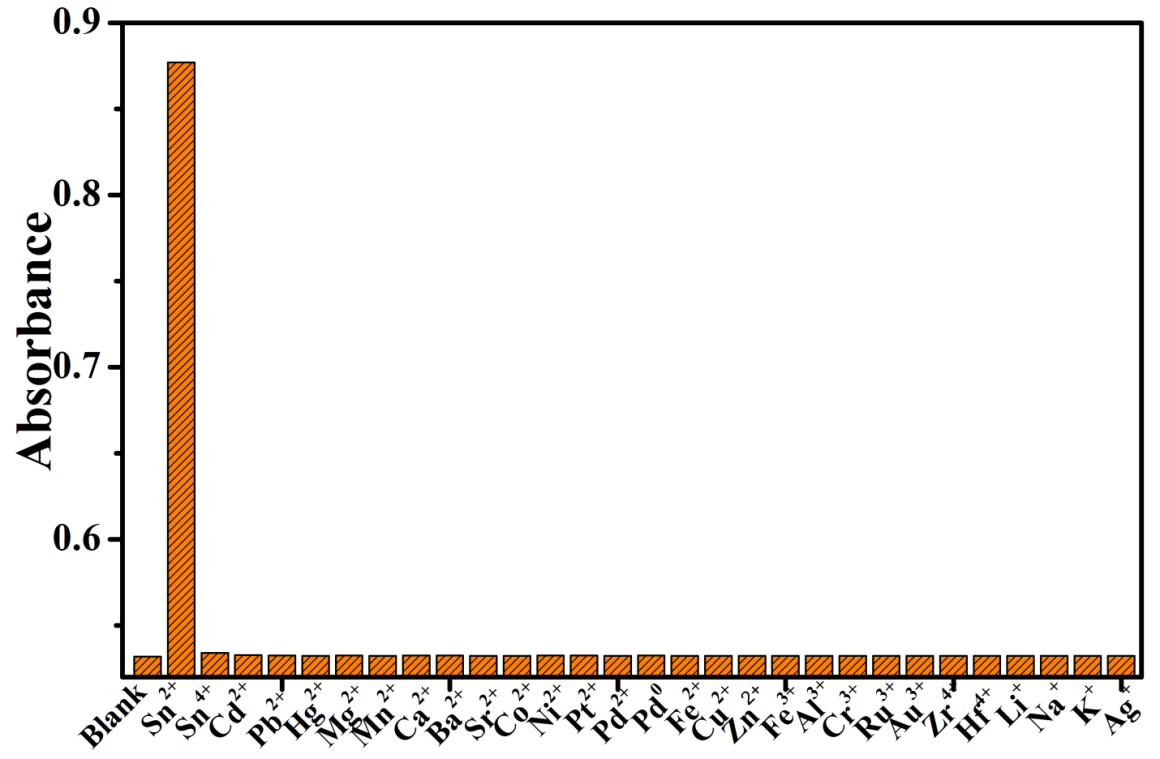


**Figure S10.** Histogram representing competitive absorbance spectra of **CNP** with various metal ions at 454 nm in H_2_O:CH_3_CN (8:1, v/v) at neutral pH (pH 7.0, 10 mM phosphate buffer).

**10. Details of Energy Calculations Using Density Functional Theory (DFT)**

Binding of **CNP** with Sn^2+^ has been investigated by quantum chemical calculations at the DFT level LANL2DZ/6-31G** method basis set implemented at Gaussian 09 program. Solvent effects were incorporated using CPCM solvent model.

^
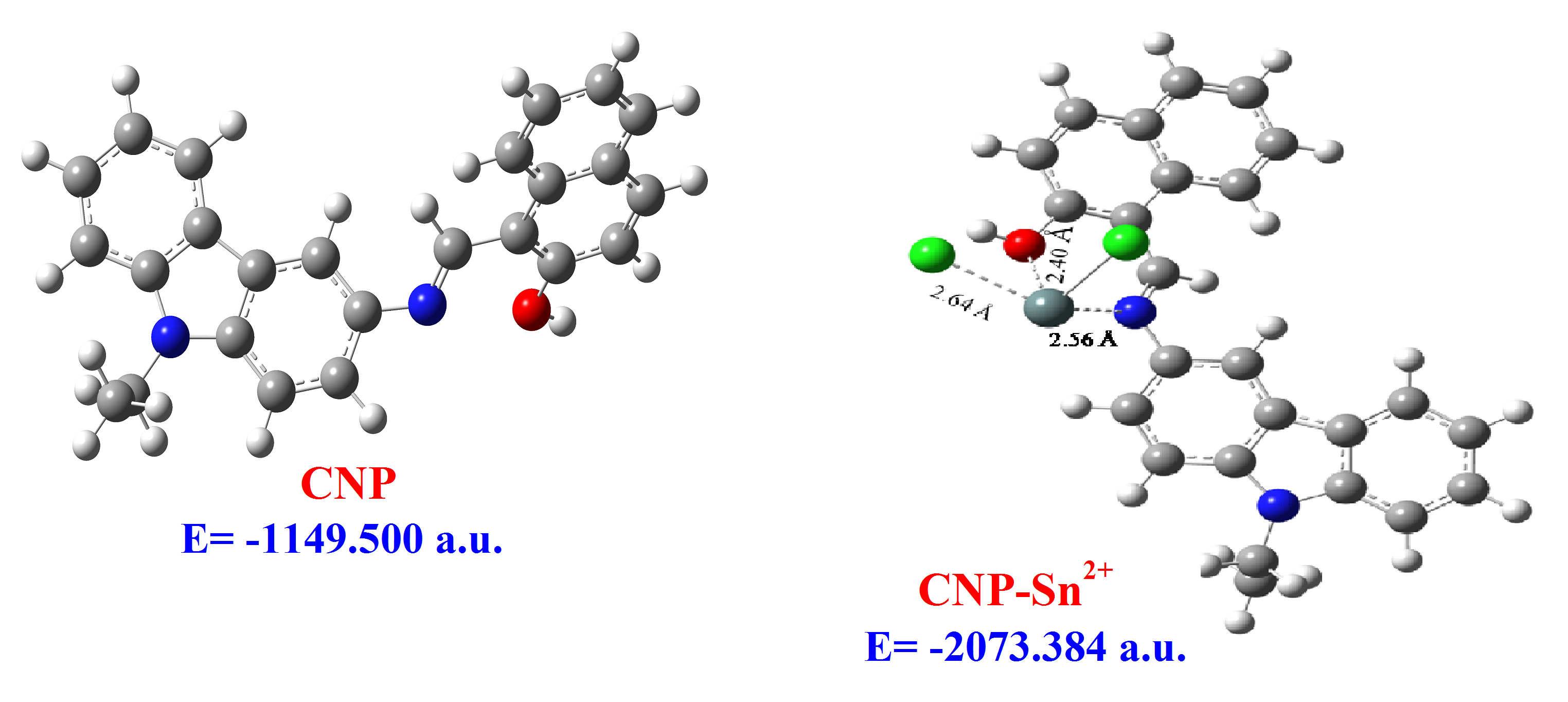
^

**Figure S11.** Energy optimized geometries of **CNP** and **CNP-Sn^2+^** complex obtained at the LANL2DZ/6-31G** levels of theory with CPCM solvation (H_2_O).

**Table S1.** Details of the geometry optimization in Gaussian 09 program.

| **Details** | **CNP** | **CNP-Sn^2+^** |
| --- | --- | --- |
| **Calculation method** | B3LYP | B3LYP |
| **Basis set** | 6-31G** | 6-31G** |
| **E(CAM-B3LYP) (a.u.)** | -1149.500 | -2073.384 |
| **Charge, Multiplicity** | 0, 1 | 0,1 |
| **Solvent (CNPCM)** | Water | Water |

**Table S2.** Selected electronic excitation energies (eV), oscillator strengths (f), main configurations of the low-lying excited states of all the molecules and complexes. The data were calculated by TDDFT//B3LYP/6-31G(d,p) based on the optimized ground state geometries.

| Molecules | Electronic Transition | Excitation  Energy^a^ | f^b^ | Composition^c^ (%) |
| --- | --- | --- | --- | --- |
|  | | | | |
| **CNP** | S_0_ → S_1_ | 3.0226 eV 410.19 nm | 0.5188 | H → L (69.6%) |
|  | S_0_ → S_5_ | 4.0902 eV 303.12 nm | 0.1057 | H → L+3 (11%) |
| **[CNP-Sn^2+^]** | S_0_ → S_1_ | 2.7637 eV 448.62nm | 0.3563 | H → L (70.2%) |
|  | S_0_ → S_2_ | 3.2342 eV 383.36 nm | 0.0589 | H-1 → L (21%) |

^a^Only selected excited states were considered. The numbers in parentheses are the excitation energy in wavelength. ^b^Oscillator strength. ^c^H stands for HOMO and L stands for LUMO.

**Table S3.** Energies of the highest occupied molecular orbital (HOMO) and lowest unoccupied

molecular orbital (LUMO)

| **Species** | **E_HOMO_ (a.u.)** | | **E_LUMO_ (a.u.)** | **∆E(a.u.)** | | **∆E(eV)** | | **∆E(kcal/mol)** |
| --- | --- | --- | --- | --- | --- | --- | --- | --- |
| **CNP** | -0.19108 | | -0.06231 | 0.12877 | | 3.50 | | 80.80 |
| **[CNP-Sn^2+^]** | -0.20265 | -0.08466 | | 0.11799 | 3.21 | | 74.04 | |

**11. Partial HRMS of the mixed assay system**

**
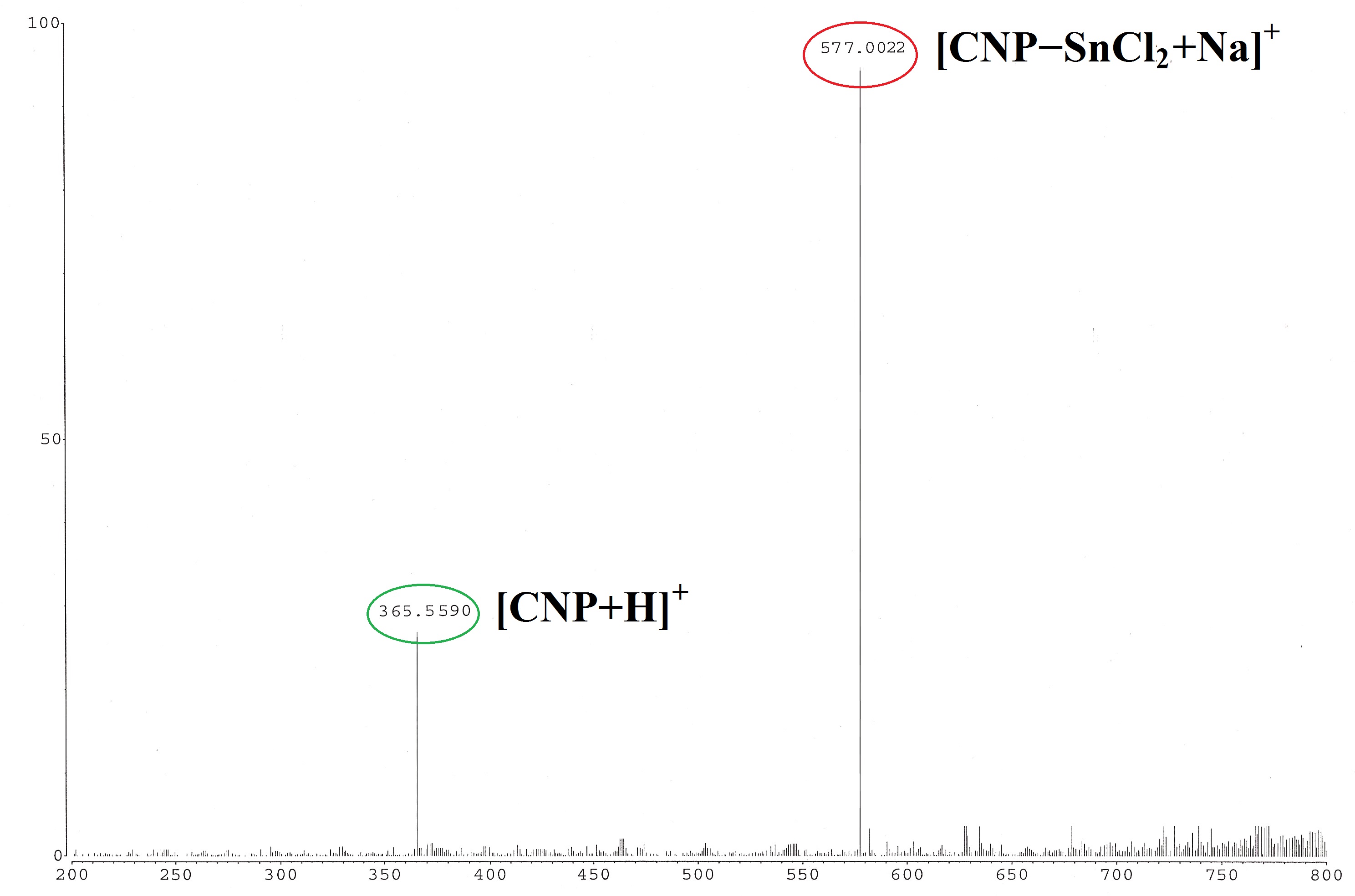
**

**Figure S12.** Partial HRMS spectra of [**CNP-Sn^2+^]** mixture in acetonitrile.

**12. IR spectroscopy**

**
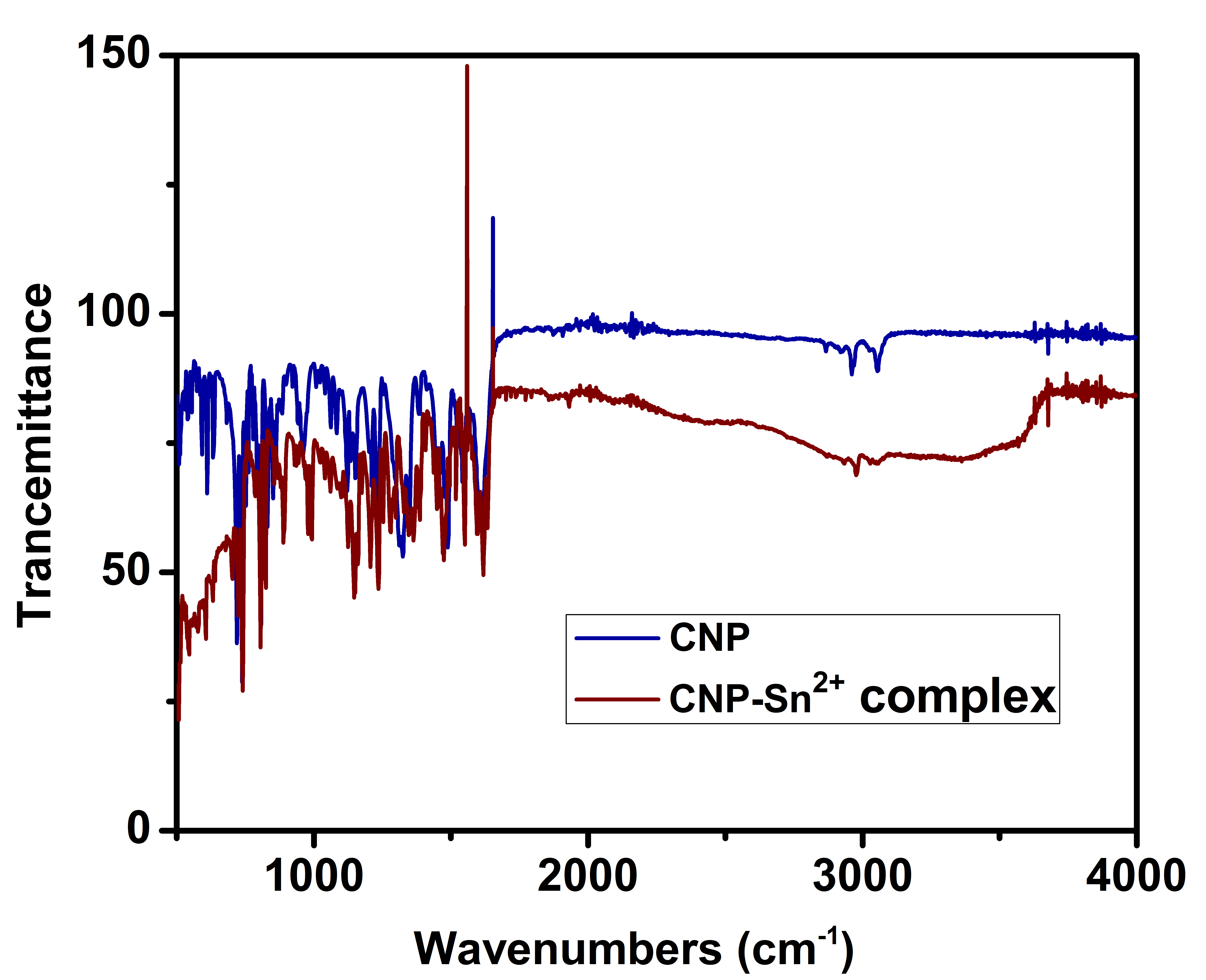
**

**Figure S13.** FT-IR spectrum of CNP and CNP-Sn^2+^ complex

**13. Preparation of Toothpaste Solution**

Commercially available toothpastes from three different brands (T1, T2, T3) were taken for this experiment.0.1g of each toothpaste sample was dissolved in 3 ml double distilled water. Next, this mixture was filtered and the filtrate was treated as toothpaste solution. The toothpaste solutions from different toothpaste samples were subjected to colorimetric analysis at pH 7.0 (10 mM phosphate buffer) to estimate the amount of Sn^2+^ present therein.

**14. Reference**

1. MERCURY (version 4.2.0). Program Package for 3D structure visualization of crystal structures, exploration of crystal packing and statistical analysis of CSD search data, **2019**.
2. L. Long, D. Zhang, X. Li, J. Zhang, C. Zhang, L. Zhou, *Anal. Chim. Acta.* **2013**, *775*,100-105.
3. M. Zhu, M. Yuan, X. Liu, J. Xu, J. Lv, C. Huang, H. Liu, Y. Li, S. Wang, D. Zhu, *Org. Lett.* **2008**, *10,*1481-1484.
